# A history of repetitive cesarean section is a risk factor of anemia in healthy perimenopausal women: The Korea National Health and Nutrition Examination Survey 2010-2012

**DOI:** 10.1371/journal.pone.0188903

**Published:** 2017-11-30

**Authors:** Jee Yoon Park, Sung Woo Lee

**Affiliations:** 1 Department of Obstetrics and Gynecology, Seoul National University Bundang Hospital, Seongnam, Korea; 2 Department of Internal Medicine, Seoul National University Postgraduate School, Seoul, Korea; 3 Department of Nephrology, Internal Medicine, Nowon Eulji Medical center, Eulji University, Seoul, Korea; National Academy of Medical Sciences, NEPAL

## Abstract

**Background and objectives:**

To determine whether the delivery method is associated with the rate of anemia in later life, using the data from the Korea National Health and Nutrition Examination Survey (KNHANES).

**Methods:**

This study used data from the KNHANES 2010–2012. Among 25,534 participants, 8,126 cases were included in this study after exclusion of males and other inappropriate data. The study population was divided into three groups according to the delivery modes: vaginal delivery (VD) only group, single cesarean delivery (SCD) group and repetitive cesarean delivery (RCD) group. The primary outcome was anemia and iron deficiency. Anemia was defined as hemoglobin <12 g/dl in accordance with the World Health Organization criteria. Iron deficiency was defined as low transferrin saturation (TSAT) or ferritin levels. Multivariate analysis was used for determination of association between the delivery modes and anemia.

**Results:**

The mean age was 53.4 years and the median time from the last delivery to the survey was 25 years. The VD only group was composed of 6,493 (79.9%) women, while 685 (8.4%) were classified as the SCD group and 948 (11.7%) were classified as a RCD group. The rates of anemia were 11.8%, 13.9%, and 19.7% in VD only group, SCD group, and RCD group, respectively. However, those groups were significantly different in many confounding factors. Therefore, to adjust those factors, multivariate analysis and subgroup analysis were followed. The odds of SCD for anemia and iron deficiency were not different from those of VD only. However, RCD was independently associated with anemia [Odds ratio(OR) 1.47, 95% Confidence interval (CI) 1.21–1.79, *P* <0.001] and iron deficiency (OR 1.42, 95% CI 1.21–1.67, *P* <0.001) compared to VD only. In the subgroup analysis, RCD was significantly associated with anemia in perimenopausal women, women with iron deficiency, those without any comorbidity and those without anemia-prone treatment.

**Conclusion:**

Repetitive cesarean section may be a risk factor for future development of anemia in later life, especially in perimenopausal period. Therefore, evaluation and management of anemia in longer postpartum period should be considered for women who experience repetitive cesarean section.

## Introduction

Although anemia is a benign condition, it largely affects the quality of life and is associated with various morbidities including cardiovascular complications [1, 2], renal diseases [3, 4], and cognitive impairment [5, 6]. The prevalence of anemia in general population worldwide was 24.8% and iron deficiency was the most common cause [7, 8]. It is continuously considered as an important issue of public health in women since the incidence and degree of disease vary throughout the life span. The National Health Insurance Service-National Health Screening Cohort database from 2002 to 2003 reported the overall prevalence of anemia in women was 15.9%, which was higher than men (5.6%) [9].

The study also demonstrated a bimodal distribution of anemia in women with peaks in age group 40–49 and over 80 years. About 20% of women experience excessive menstrual blood loss in their 40s and most of them are caused by bleeding abnormalities associated with uterine fibroids or anovulation [10]. Two thirds of women with menorrhagia have iron deficiency anemia [11]. One third of anemia in elderly is from nutritional deficiency and often associated with gastrointestinal bleeding or chronic diseases such as kidney diseases and other inflammation [12–14].

Pregnancy is a well-known risk factor of iron deficiency anemia in women. During pregnancy, plasma volume and red blood cell mass simultaneously increase and hemodilution is resulted [15]. This phenomenon develops physiologic anemia and increased demand on iron due to the need for fetal growth leads to further depletion of maternal iron stores [16, 17]. Postpartum anemia is commonly followed because of blood loss at delivery and preexisting iron deficiency during antepartum period [18, 19]. The incidence of anemia in 6 months after delivery reported ranges from 12.7% to 30% [20–22].

The average estimated blood loss at the time of delivery is about 500 mL [23]. The amount of blood loss during delivery of a single baby The average amount of blood loss after the birth of a single baby varies according to the individual differences in coagulation profiles, race, parity, placental abnormalities and the quality of obstetric facilities, however it is usually considered that the blood loss during vaginal delivery is considerably less than that during cesarean section [18]. The natural course of recovery from postpartum anemia has never been researched and indefinite duration of recommendation on oral iron supplementations is recommended during puerperium and period of breastfeeding. Moreover, the data on the influence of delivery mode to the development of anemia in long-term follow-ups are lacking.

This study aims to determine whether the delivery method is associated with the rate of anemia in later life, using the data from the Korea National Health and Nutrition Examination Survey (KNHANES).

## Material and methods

### Participants

The KNHANES has been performed periodically since 1998 to assess the health and nutritional status of the civilian in non-institutionalized Korean population. Participants were selected using proportional allocation systematic sampling with multistage stratification. This study used data from the KNHANES 2010–2012. Of 31,596 candidates, 25,534 people agreed to participate the KNHANES 2010–2012 (participating rate: 71.3%). The study protocol complied with the Declaration of Helsinki. Full approval of the study was obtained from the Institutional Review Board of the Korea Centers for Disease Control (IRB number: 2010-02CON-21-C, 2011-02CON-06-C, 2012-01EXP-01-2C). All data were fully anonymized before we accessed them. KNHANES participants provided informed written consent to have their data used in research. The protocol comprised a health-questionnaire survey, health examination, and nutrition survey.

Among 25,534 participants, we excluded 5,935 cases aged <19 years first. Of 19,599 adults aged ≥19 years, 8,461 men, 849 missing menstrual information, 8 missing exogenous estrogen exposure history, 38 missing obstetric information, 636 missing data regarding diagnostic studies on anemia [hemoglobin, transferrin saturation (TSAT), and ferritin], and 1,481 who have never been pregnant were excluded. This study therefore included 8,126 participants (S1 Fig).

### Data collection and measurements

A standardized interview was conducted in the homes of the participants to collect information including demographic variables, obstetrics and gynecologic history, medical history, medications used, and a variety of other health-related variables. Blood pressure (BP) was measured three times in accordance with the standard protocol, and mean values of the three measures were used as the representative BP. Height was measured to the nearest 0.1 cm using a portable stadiometer (Seriter, Bismarck, ND). Weight was measured to the nearest 0.1 kg using a Giant-150N calibrated balance-beam scale (Hana, Seoul, Korea). Body mass index (BMI) was calculated by dividing the weight by the square of the height (kg/m^2^).

Blood samples were collected in the morning after at least 8 hours`fast and analyzed at central laboratory (Neodin Medical Institute, Seoul, Korea). The hemoglobin level was measured via the cyanide-free sodium lauryl sulphate method using a XE-2100D (Sysmex, Kobe, Japan). Serum iron and ferritin levels were measured by a Bathophenanthroline direct method with a Hitachi Automatic Analyzer 7600 (Hitachi, Tokyo, Japan) and an immunoradiometric assay with a 1470 WIZARD gamma counter (PerkinElmer,Waltham, MA, USA), respectively. TSAT (%) was calculated as serum iron × 100/total iron binding capacity.

Anemia was defined as hemoglobin <12 g/dl in accordance with the World Health Organization criteria. The definition of anemia-prone treatment was the ongoing treatment course of cardiovascular disease or arthritis. Iron deficiency was defined as low TSAT or ferritin levels. Since there is no agreed consensus of cut-offs for the low TSAT and ferritin levels in diagnosis of iron deficiency [24], we defined low TSAT and ferritin levels as lower serum levels of TSAT and ferritin than the respective best cut-offs for the association with anemia in our study participants. When cesarean delivery (CD) was performed only once in lifetime, she was classified as single CD (SCD) group. Repetitive CD (RCD) group was defined as women who had history of two or more times of cesarean sections. When a woman had both vaginal delivery (VD) and CD, she was assigned to CD groups regardless of the sequence of delivery modes.

### Statistical analysis

The distributions of continuous variables were evaluated using histograms and Q-Q plots. Normally distributed continuous variables are expressed as mean ± standard deviation (SD), non-normally distributed continuous variables as median (interquartile range), and categorical variables as percentages. Difference was analyzed by one-way ANOVA for normally distributed continuous variables, Kruskal-Wallis test for non-normally distributed continuous variables, and a chi-square test for categorical variables. Post-hoc analysis was performed using Bonferroni method of one-way ANOVA for normally distributed continuous variables, Mann-Whitney U tests for non-normally distributed continuous variables, and chi-square tests for categorical variables.

The relationship between TSAT and ferritin and anemia was plotted using the penalized smoothing spline method with the “pspline” package in R Statistics (version 3.03). The area under the curve (AUC) with its 95% confidence interval (CI) of receiver operating characteristic curve (ROC) was evaluated using R Statics (version 3.03) with “pROC” packages. The best cut-offs was calculated by obtaining the Youden index (sensitivity + specificity—1). Odds ratio and its 95% CI were calculated using logistic regression analysis.

In multivariate analysis, covariates were chosen based on clinical relevance: age, comorbidity (hypertension, diabetes, dyslipidemia, cancer history, treatment of cardiovascular disease and arthritis, and BMI), smoking and drinking status, white blood cells counts, obstetric and gynecologic variables (menopause, numbers of pregnancy and abortion, and exogenous estrogen exposure). A *P*-value of <0.05 was considered statistically significant. All analyses unless otherwise specified were performed using SPSS Version 22 (IBM Corp. released 2013, Armonk, NY).

## Results

In the total study population, the mean age was 53.4 years and the median numbers of delivery was two. The median time from the last delivery to the survey was 25 years. At the time of the survey, 57.2% women were at menopause. The VD only group was the largest and was composed of 6,493 (79.9%) women, while 685 (8.4%) were classified as the SCD group and 948 (11.7%) were classified as a RCD group. The rate of anemia was 12.9%. Mean ± SD value of TSAT was 32.3 ± 13.6% and median (interquartile range) of ferritin was 93.3 (47.9–157.6) *p*mol/l. The proportion of oral pills usage was 17.2% and the history of estrogen replacement treatment was found in 10.3%. The rates of hypertension, diabetes, dyslipidemia, and cancer history were 33.8%, 10.0%, 21.6%, and 4.1%, respectively.

**Fig 1:**
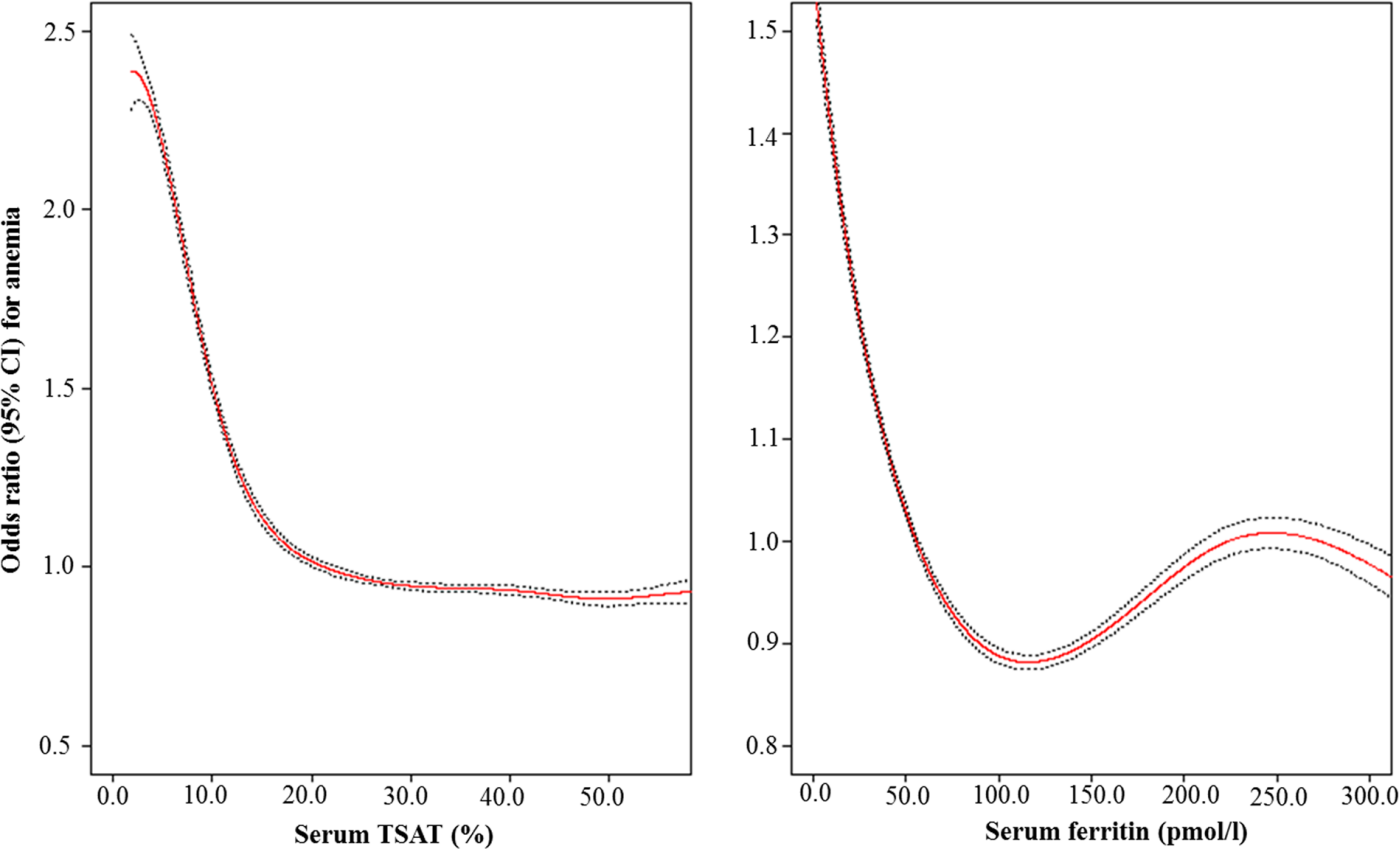
Penalized smoothing splines showing the relationship between TSAT, ferritin and anemia. CI, confidence interval; TSAT, transferrin saturation. The upper 5% of the TSAT and ferritin were truncated. The red line indicated the odds ratio and the black dotted line indicated the 95% CI for which TSAT and ferritin influence the anemia.

**Fig 2:**
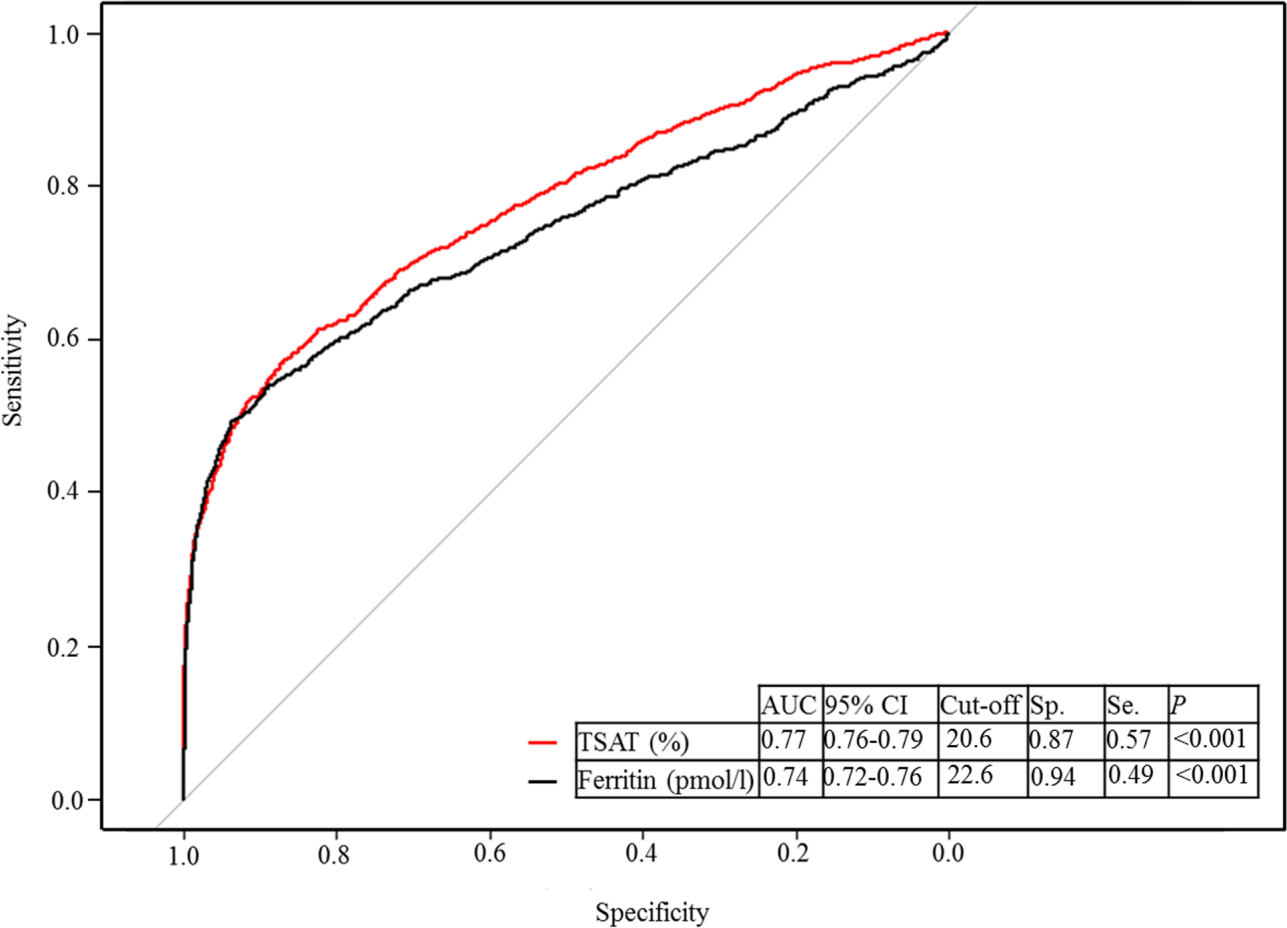
Receiver operating characteristics analysis of TSAT and ferritin for anemia. AUC, area under the curve; CI, confidence interval; Se, sensitivity; Sp, specificity; TSAT, transferrin saturation.

We plotted the relationships between TSAT, ferritin, and anemia using the penalized smoothing spline analysis (Fig 1). TSAT showed L-shaped association with anemia, whereas ferritin revealed U-shaped association with anemia. Since the relationships between TSAT, ferritin and anemia were non-linear, we performed ROC analysis to identify best cut-offs of TSAT and ferritin for the association with anemia (Fig 2). In the analysis, 20.6% of TSAT and 22.6 *p*mol/l of ferritin were found to be associated with anemia best. The rates of low TSAT and ferritin levels defined as TSAT <20.6% and ferritin <22.6 *p*mol/l were 18.3% and 11.9%, respectively, and the rate of iron deficiency was 21.9% accordingly.

The clinical characteristics according to the delivery modes are demonstrated (Table 1). Women in SCD and RCD groups were younger than those in VD only group. More women in VD only group were at menopause and had an anemia-prone treatment than those in SCD and RCD groups. The proportions of never smokers and never drinkers were both significantly higher in VD only group than in SCD and RCD groups. The rates of hypertension, diabetes and dyslipidemia were higher in VD only group than other groups. Women in RCD group showed higher rate of never smoker and lower rates of hypertension, diabetes, dyslipidemia, anemia-prone treatment, and menopause than those in SCD group despite of similar age. Compared to women in VD only group, those in SCD and RCD groups showed significantly higher rate of iron deficiency. Women with RCD were more iron-deficient than those with SCD. Although the rates of anemia in VD only group and in SCD group were comparable, women in RCD group revealed significantly higher rate of anemia than those in other groups.

**Table 1:** Clinical characteristics according to the delivery modes.

	VD only (n = 6493)	SCD (n = 685)	RCD (n = 948)	*P*
Age (years)	56.1 ± 14.1	42.9 ± 10.6*	42.4 ± 7.8*	<0.001
Numbers of pregnancy (no.)	4.0 (3.0–6.0)	2.0 (1.0–4.0)*	3.0 (2.0–4.0)*†	<0.001
Abortion ≥3 times (%)	19.3	13.3*	9.1*†	<0.001
Delivery ≥3 times (%)	47.7	14.6*	15.5*	<0.001
Time from last delivery (years)	28.0 (15.0–36.0)	10.0 (3.0–18.0)*	10.0 (5.0–16.0)*	<0.001
Menopause (%)	66.2	25.7*	18.2*†	<0.001
Exogenous estrogen (%)	26.4	17.8*	13.3*†	<0.001
Never smoker (%)	92.5	82.8*	88.4*†	<0.001
Never drinker (%)	25.3	10.7*	10.0*	<0.001
Hypertension (%)	38.7	17.4*	12.5*†	<0.001
Diabetes (%)	11.4	5.8*	3.7*†	<0.001
Dyslipidemia (%)	24.0	15.1*	10.6*†	<0.001
Cancer history (%)	4.3	3.5	3.2	0.184
Anemia-prone treatment (%)	18.7	6.6*	3.4*†	<0.001
CVD treatment (%)	3.5	1.5*	0.2*†	<0.001
Arthritis treatment (%)	16.1	5.4*	3.2*†	<0.001
BMI (kg/m^2^)	23.8 ± 3.3	23.6 ± 3.7	23.6 ± 3.6	0.334
Glucose (mmol/l)	5.4 ± 1.2	5.2 ± 1.1*	5.2 ± 1.1*	<0.001
Cholesterol (mmol/l)	5.1 ± 1.0	4.9 ± 1.0*	4.9 ± 0.9*	<0.001
WBC(x10^3^/μL)	5.7 ± 1.6	5.8 ± 1.6	5.6 ± 1.5	0.249
Hemoglobin (g/dl)	13.1 ± 1.1	13.0 ± 1.2	12.8 ± 1.3*†	<0.001
Anemia (%)	11.8	13.9	19.7*†	<0.001
TSAT (%)	32.7 ± 13.1	32.2 ± 15.3	30.1 ± 15.9*†	<0.001
Ferritin (pmol/l)	101.8 (55.5–167.0)	73.3 (33.6–128.2)*	55.5 (21.9–104.3)*†	<0.001
Low TSAT levels (%)	16.2	22.0*	30.1*†	<0.001
Low Ferritin levels (%)	9.4	16.2*	25.6*†	<0.001
Iron deficiency (%)	19.4	26.4*	36.1*†	<0.001

VD, vaginal delivery; SCD, single cesarean delivery; RCD, repetitive cesarean delivery; CVD, cardiovascular disease; BMI, body mass index; WBC, white blood cells; TSAT, transferrin saturation. Values are expressed as mean ± standard deviation for normally distributed continuous variables, median (interquartile range) for non-normally distributed continuous variables, and percentage for categorical variables. Difference was analyzed by one-way ANOVA for normally distributed continuous variables, Kruskal-Wallis test for non-normally distributed continuous variables, and a chi-square test for categorical variables.

* and †meant *P* < 0.05 when compared to VD only and SCD, respectively, by using Bonferroni post-hoc analysis of one-way ANOVA for normally distributed continuous variables, Mann-Whitney U test for non-normally distributed continuous variables, and chi-square test for categorical variables.

To identify the association of delivery modes with anemia and iron deficiency, multivariate logistic regression analysis was performed (Table 2). The odds of SCD for anemia and iron deficiency were not different from those of VD only. However, RCD was independently associated with 1.472-times (95% CI 1.212–1.789, *P* <0.001) higher odds for anemia and 1.422-times (95% CI 1.211–1.669, *P* <0.001) higher odds for iron deficiency than VD only. In the subgroup analysis, RCD was significantly associated with increased odds for anemia in cases before menopause, those with iron deficiency, those without any comorbidity and those without anemia-prone treatment (Table 3).

**Table 2:** Odds ratio of delivery group for the anemia and iron deficiency.

	Anemia		Iron deficiency	
Delivery group	Adjusted OR (95% CI)	*P*	Adjusted OR (95% CI)	*P*
Single CD vs. VD only	1.153 (0.901–1.474)	0.257	1.046 (0.860–1.271)	0.654
Repetitive CD vs. VD only.	1.472 (1.212–1.789)	<0.001	1.422 (1.211–1.669)	<0.001

OR, odds ratio; CI, confidence interval; VD, vaginal delivery; CD, cesarean delivery. Rows were factor and columns were outcome. Adjusted OR and 95% CI were analyzed using multivariate logistic regression analysis entering into age, hypertension, diabetes, dyslipidemia, cancer history, cardiovascular disease treatment, arthritis treatment, body mass index, smoking and drinking status, white blood cells counts, menopause, numbers of pregnancy and abortion, and exogenous estrogen exposure as covariates.

**Table 3:** Subgroup analysis of the association between delivery group and anemia.

Subgroup		Delivery group	Adjusted OR (95% CI)	*P*
Menopause	No (n = 3477)	Single CD vs. VD only	1.060 (0.799–1.404)	0.687
		Repetitive CD vs. VD only	1.520 (1.236–1.869)	<0.001
	Yes (n = 4649)	Single CD vs. VD only	1.429 (0.814–2.509)	0.213
		Repetitive CD vs. VD only	0.635 (0.273–1.480)	0.293
Iron deficiency	No (n = 6344)	Single CD vs. VD only	0.940 (0.587–1.506)	0.798
		Repetitive CD vs. VD only	0.893 (0.574–1.387)	0.614
	Yes (n = 1782)	Single CD vs. VD only	1.234 (0.874–1.743)	0.232
		Repetitive CD vs. VD only	1.356 (1.040–1.768)	0.025
Comorbidity	No (n = 4249)	Single CD vs. VD only	1.143 (0.855–1.528)	0.367
		Repetitive CD vs. VD only	1.537 (1.237–1.909)	<0.001
	Yes (n = 3854)	Single CD vs. VD only	1.175 (0.725–1.903)	0.513
		Repetitive CD vs. VD only	1.156 (0.728–1.836)	0.539
Anemia-prone Tx	No (n = 6826)	Single CD vs. VD only	1.135 (0.880–1.463)	0.329
		Repetitive CD vs. VD only	1.443 (1.184–1.759)	<0.001
	Yes (n = 1290)	Single CD vs. VD only	1.108 (0.397–3.094)	0.844
		Repetitive CD vs. VD only	1.711 (0.569–5.142)	0.339

OR, odds ratio; CI, confidence interval; VD, vaginal delivery; CD, cesarean delivery; Tx, treatment. Comorbidity included hypertension, diabetes, dyslipidemia, or cancer history. Adjusted OR and 95% CI were analyzed using multivariate logistic regression analysis entering into age, hypertension, diabetes, dyslipidemia, cancer history, cardiovascular treatment, arthritis treatment, body mass index, smoking and drinking status, white blood cells counts, menopause, numbers of pregnancy and abortion, and exogenous estrogen exposure as covariates.

## Discussion

The principal finding of this study was that the history of repetitive cesarean section was independently associated with both anemia and iron deficiency in later life. Since the clinical characteristics were not comparable among the three groups divided according to the delivery modes in the past, subgroup analysis was followed. The association between repetitive cesarean section and anemia was evident in women before menopause, those who showed laboratory findings of iron deficiency, those without any comorbidity and those who had never received anemia-prone treatment. In all aspects of analysis, the risks of anemia in women with the experience of cesarean section only once were not significantly different from those in women who had undergone vaginal delivery only.

The relationship between anemia and cesarean section has been described before, however the causal relationship was reversed compared to this study. Previous studies reported mothers with antepartum anemia are at increased risk of cesarean section [25–31]. Some suggested that maternal anemia tended to cause fetal distress and subsequently increase the cesarean section rate [31–33]. Indeed, preexisting iron deficiency anemia in pregnancy has been reported to be associated with adverse pregnancy outcomes such as premature birth and low birth weight [34].

The repetitive cesarean section during reproductive age as a cause of anemia in later life can be explained by two hypotheses. First, the average amount of blood loss caused by cesarean section is about twice higher than VD [35]. The more excessive blood loss may not be compensated in about 25 years, which was the median value of time period from the last delivery to the survey in this study population. Jasen et al. reported that the expected drop in hemoglobin of less than 0.6 g/dL at 3 days after VD is quickly recovered by postpartum diuresis which eliminates the excess plasma volume and leads to hemoconcentration [36]. The study described that hemoglobin generally returns to normal values in one month after VD [36]. The data on recovery pattern of hemoglobin after cesarean section are lacking.

Secondly, the recovery on physical activity performance is faster in women after VD than those after CD. Since cesarean section is a type of surgery, it results in more pain, needs more time to recover from the anesthesia, and causes more complications. The decreased exercise has been suggested as a risk factor for aggravation of anemia in geriatric patients [37, 38].

The subgroup analysis performed on the association with anemia according to the various confounding factors demonstrated that the history of RCD increased the development of anemia only before menopause. Once women end up the reproductive period and enter menopause, anemia becomes even less prevalent [39]. The regular blood loss caused by menstruation seems to be a critical factor for the development of anemia during reproductive age. In women with iron deficiency, the effect of repetitive cesarean section on anemia compared to VD was definite after adjusting with other factors, probably due to the depletion of iron storage.

In addition, among women with relatively healthier condition such as no comorbidity and no anemia-prone treatment, the association between repetitive cesarean section and anemia was significant. This must be because the presence of current comorbidity or other chronic diseases supplied by anemia-prone treatment are far stronger risk factors for the development of anemia and the degree of iron deficiency than the history of cesarean section about 20 years ago.

This study is unique since the data on the association between current health condition and previous obstetric histories are scarce. Nevertheless, certain limitations are inevitable. Since the study design was cross-sectional, the causal relationship between obstetric histories and the currently diagnosed anemia suggested in this study might not be strongly convincing. Furthermore, the survey relied on the history taking of obstetric events in the past, the recall bias cannot be excluded.

There are a few studies followed-up the status of anemia in postpartum period. Milman et al. demonstrated that there was a remarkable decline in serum iron during the first week of postpartum period and it started to increase from 1 to 8 weeks postpartum [40]. However observation of anemia caused by delivery in long-term period is extremely difficult. Therefore, the result in this study could be a beginning step to find the association between anemia in later life and past histories of pregnancy. Future prospective studies in long-term follow-ups are necessary.

In conclusion, repetitive cesarean section may be a risk factor for future development of anemia in later life, especially in perimenopausal period. Since anemia in women of all ages affect the quality of life and diverse comorbidities, thorough concern for postpartum evaluation of anemia in longer period and active supplementation of oral iron for women who experience repetitive cesarean section must be considered.

## Supporting information

S1 Fig Flow diagram of the participants’ inclusion. (PPTX)

S1 File Dataset of this study. (XLSX)

## References

[1] Herzog CA, Muster HA, Li S, Collins AJ. Impact of congestive heart failure, chronic kidney disease, and anemia on survival in the Medicare population. J Card Fail. 2004;10(6):467–72. . 15599836 10.1016/j.cardfail.2004.03.003

[2] Lee WC, Fang HY, Chen HC, Chen CJ, Yang CH, Hang CL, et al Anemia: A significant cardiovascular mortality risk after ST-segment elevation myocardial infarction complicated by the comorbidities of hypertension and kidney disease. PLoS One. 2017;12(7):e0180165 doi: 10.1371/journal.pone.0180165 ; PubMed Central PMCID: PMCPMC5531517. 28749948 10.1371/journal.pone.0180165 PMC5531517

[3] Metivier F, Marchais SJ, Guerin AP, Pannier B, London GM. Pathophysiology of anaemia: focus on the heart and blood vessels. Nephrol Dial Transplant. 2000;15 Suppl 3:14–8. . 11032352 10.1093/oxfordjournals.ndt.a027970

[4] Stauffer ME, Fan T. Prevalence of anemia in chronic kidney disease in the United States. PLoS One. 2014;9(1):e84943 doi: 10.1371/journal.pone.0084943 ; PubMed Central PMCID: PMCPMC3879360. 24392162 10.1371/journal.pone.0084943 PMC3879360

[5] Petranovic D, Batinac T, Petranovic D, Ruzic A, Ruzic T. Iron deficiency anaemia influences cognitive functions. Med Hypotheses. 2008;70(1):70–2. doi: 10.1016/j.mehy.2007.04.029 . 17574345 10.1016/j.mehy.2007.04.029

[6] Lucca U, Tettamanti M, Mosconi P, Apolone G, Gandini F, Nobili A, et al Association of mild anemia with cognitive, functional, mood and quality of life outcomes in the elderly: the "Health and Anemia" study. PLoS One. 2008;3(4):e1920 doi: 10.1371/journal.pone.0001920 ; PubMed Central PMCID: PMCPMC2271152. 18382689 10.1371/journal.pone.0001920 PMC2271152

[7] McLean E, Cogswell M, Egli I, Wojdyla D, de Benoist B. Worldwide prevalence of anaemia, WHO Vitamin and Mineral Nutrition Information System, 1993–2005. Public Health Nutr. 2009;12(4):444–54. doi: 10.1017/S1368980008002401 . 18498676 10.1017/S1368980008002401

[8] Lopez A, Cacoub P, Macdougall IC, Peyrin-Biroulet L. Iron deficiency anaemia. Lancet. 2016;387(10021):907–16. doi: 10.1016/S0140-6736(15)60865-0 . 26314490 10.1016/S0140-6736(15)60865-0

[9] Seong SC, Kim YY, Park SK, Khang YH, Kim HC, Park JH, et al Cohort profile: the National Health Insurance Service-National Health Screening Cohort (NHIS-HEALS) in Korea. BMJ Open. 2017;7(9):e016640 doi: 10.1136/bmjopen-2017-016640 ; PubMed Central PMCID: PMCPMC5623538. 28947447 10.1136/bmjopen-2017-016640 PMC5623538

[10] Chen BH, Giudice LC. Dysfunctional uterine bleeding. West J Med. 1998;169(5):280–4. ; PubMed Central PMCID: PMCPMC1305317. 9830356 PMC1305317

[11] Cohen BJ, Gibor Y. Anemia and menstrual blood loss. Obstet Gynecol Surv. 1980;35(10):597–618. . 6997784

[12] Patel KV. Epidemiology of anemia in older adults. Semin Hematol. 2008;45(4):210–7. doi: 10.1053/j.seminhematol.2008.06.006 ; PubMed Central PMCID: PMCPMC2572827. 18809090 10.1053/j.seminhematol.2008.06.006 PMC2572827

[13] Kanuri G, Sawhney R, Varghese J, Britto M, Shet A. Iron Deficiency Anemia Coexists with Cancer Related Anemia and Adversely Impacts Quality of Life. PLoS One. 2016;11(9):e0163817 doi: 10.1371/journal.pone.0163817 ; PubMed Central PMCID: PMCPMC5040456. 27682226 10.1371/journal.pone.0163817 PMC5040456

[14] Silva EC, Roriz AK, Eickemberg M, Mello AL, Cortes EB, Feitosa CA, et al Factors Associated with Anemia in the Institutionalized Elderly. PLoS One. 2016;11(9):e0162240 doi: 10.1371/journal.pone.0162240 ; PubMed Central PMCID: PMCPMC5015845. 27607057 10.1371/journal.pone.0162240 PMC5015845

[15] Sifakis S, Pharmakides G. Anemia in pregnancy. Ann N Y Acad Sci. 2000;900:125–36. . 10818399 10.1111/j.1749-6632.2000.tb06223.x

[16] Routine iron supplementation during pregnancy. Review article. US Preventive Services Task Force. JAMA. 1993;270(23):2848–54. . 8133626

[17] Routine iron supplementation during pregnancy. Policy statement. US Preventive Services Task Force. JAMA. 1993;270(23):2846–8. . 8133625

[18] Milman N. Postpartum anemia I: definition, prevalence, causes, and consequences. Ann Hematol. 2011;90(11):1247–53. doi: 10.1007/s00277-011-1279-z . 21710167 10.1007/s00277-011-1279-z

[19] Milman N. Anemia—still a major health problem in many parts of the world! Ann Hematol. 2011;90(4):369–77. doi: 10.1007/s00277-010-1144-5 . 21221586 10.1007/s00277-010-1144-5

[20] Bodnar LM, Cogswell ME, McDonald. Have we forgotten the significance of postpartum iron deficiency? Am J Obstet Gynecol. 2005;193(1):36–44. doi: 10.1016/j.ajog.2004.12.009 . 16021056 10.1016/j.ajog.2004.12.009

[21] Corwin EJ, Murray-Kolb LE, Beard JL. Low hemoglobin level is a risk factor for postpartum depression. J Nutr. 2003;133(12):4139–42. . 14652362 10.1093/jn/133.12.4139

[22] Becuzzi N, Zimmermann R, Krafft A. Long-term efficacy of postpartum intravenous iron therapy. Biomed Res Int. 2014;2014:815437 doi: 10.1155/2014/815437 ; PubMed Central PMCID: PMCPMC4238267. 25431768 10.1155/2014/815437 PMC4238267

[23] Nash CM, Allen VM. The Use of Parenteral Iron Therapy for the Treatment of Postpartum Anemia. J Obstet Gynaecol Can. 2015;37(5):439–42. doi: 10.1016/S1701-2163(15)30259-0 . 26168105 10.1016/S1701-2163(15)30259-0

[24] Peyrin-Biroulet L, Williet N, Cacoub P. Guidelines on the diagnosis and treatment of iron deficiency across indications: a systematic review. Am J Clin Nutr. 2015;102(6):1585–94. doi: 10.3945/ajcn.114.103366 . 26561626 10.3945/ajcn.114.103366

[25] Hwang HS, Kim YH, Kwon JY, Park YW. Uterine and umbilical artery Doppler velocimetry as a predictor for adverse pregnancy outcomes in pregnant women with anemia. J Perinat Med. 2010;38(5):467–71. doi: 10.1515/JPM.2010.047 . 20443671 10.1515/jpm.2010.047

[26] Stafford I, Dildy GA, Clark SL, Belfort MA. Visually estimated and calculated blood loss in vaginal and cesarean delivery. Am J Obstet Gynecol. 2008;199(5):519 e1–7. doi: 10.1016/j.ajog.2008.04.049 . 18639209 10.1016/j.ajog.2008.04.049

[27] Levy A, Fraser D, Katz M, Mazor M, Sheiner E. Maternal anemia during pregnancy is an independent risk factor for low birthweight and preterm delivery. Eur J Obstet Gynecol Reprod Biol. 2005;122(2):182–6. doi: 10.1016/j.ejogrb.2005.02.015 . 16219519 10.1016/j.ejogrb.2005.02.015

[28] Malhotra M, Sharma JB, Batra S, Sharma S, Murthy NS, Arora R. Maternal and perinatal outcome in varying degrees of anemia. Int J Gynaecol Obstet. 2002;79(2):93–100. . 12427391 10.1016/s0020-7292(02)00225-4

[29] Hamalainen H, Hakkarainen K, Heinonen S. Anaemia in the first but not in the second or third trimester is a risk factor for low birth weight. Clin Nutr. 2003;22(3):271–5. . 12765667 10.1016/s0261-5614(02)00209-1

[30] Ehrenthal DB, Chichester ML, Cole OS, Jiang X. Maternal risk factors for peripartum transfusion. J Womens Health (Larchmt). 2012;21(7):792–7. doi: 10.1089/jwh.2011.3248 . 22500552 10.1089/jwh.2011.3248

[31] Drukker L, Hants Y, Farkash R, Ruchlemer R, Samueloff A, Grisaru-Granovsky S. Iron deficiency anemia at admission for labor and delivery is associated with an increased risk for Cesarean section and adverse maternal and neonatal outcomes. Transfusion. 2015;55(12):2799–806. doi: 10.1111/trf.13252 . 26246160 10.1111/trf.13252

[32] Park YS, Hoh JK. Complex and irregular heart rate dynamics in fetuses compromised by maternal anemia as a high-risk pregnancy. J Perinat Med. 2015;43(6):741–8. doi: 10.1515/jpm-2014-0104 . 25178901 10.1515/jpm-2014-0104

[33] Haider BA, Olofin I, Wang M, Spiegelman D, Ezzati M, Fawzi WW, et al Anaemia, prenatal iron use, and risk of adverse pregnancy outcomes: systematic review and meta-analysis. BMJ. 2013;346:f3443 doi: 10.1136/bmj.f3443 ; PubMed Central PMCID: PMCPMC3689887. 23794316 10.1136/bmj.f3443 PMC3689887

[34] Milman N. Prepartum anaemia: prevention and treatment. Ann Hematol. 2008;87(12):949–59. doi: 10.1007/s00277-008-0518-4 . 18641987 10.1007/s00277-008-0518-4

[35] American College of O, Gynecologists. ACOG Practice Bulletin: Clinical Management Guidelines for Obstetrician-Gynecologists Number 76, October 2006: postpartum hemorrhage. Obstet Gynecol. 2006;108(4):1039–47. . 17012482 10.1097/00006250-200610000-00046

[36] Jansen AJ, van Rhenen DJ, Steegers EA, Duvekot JJ. Postpartum hemorrhage and transfusion of blood and blood components. Obstet Gynecol Surv. 2005;60(10):663–71. . 16186783 10.1097/01.ogx.0000180909.31293.cf

[37] Maraldi C, Ble A, Zuliani G, Guralnik JM, Mussi C, Fellin R, et al Association between anemia and physical disability in older patients: role of comorbidity. Aging Clin Exp Res. 2006;18(6):485–92. . 17255637 10.1007/BF03324848

[38] Okonko DO, Mandal AK, Missouris CG, Poole-Wilson PA. Disordered iron homeostasis in chronic heart failure: prevalence, predictors, and relation to anemia, exercise capacity, and survival. J Am Coll Cardiol. 2011;58(12):1241–51. doi: 10.1016/j.jacc.2011.04.040 . 21903058 10.1016/j.jacc.2011.04.040

[39] Friedman AJ, Chen Z, Ford P, Johnson CA, Lopez AM, Shander A, et al Iron deficiency anemia in women across the life span. J Womens Health (Larchmt). 2012;21(12):1282–9. doi: 10.1089/jwh.2012.3713 . 23210492 10.1089/jwh.2012.3713

[40] Milman N, Agger AO, Nielsen OJ. Iron supplementation during pregnancy. Effect on iron status markers, serum erythropoietin and human placental lactogen. A placebo controlled study in 207 Danish women. Dan Med Bull. 1991;38(6):471–6. . 1802636

