# Supplementary material for: A history of repetitive cesarean section is a risk factor of anemia in healthy perimenopausal women: The Korea National Health and Nutrition Examination Survey 2010-2012
Source: PLoS One. 2017 Nov 30;12(11):e0188903. doi: 10.1371/journal.pone.0188903 (PMC5708789; doi:10.1371/journal.pone.0188903)
Supplement: S1 Fig — (PPTX) [file pone.0188903.s001.pptx]

## Slide 1
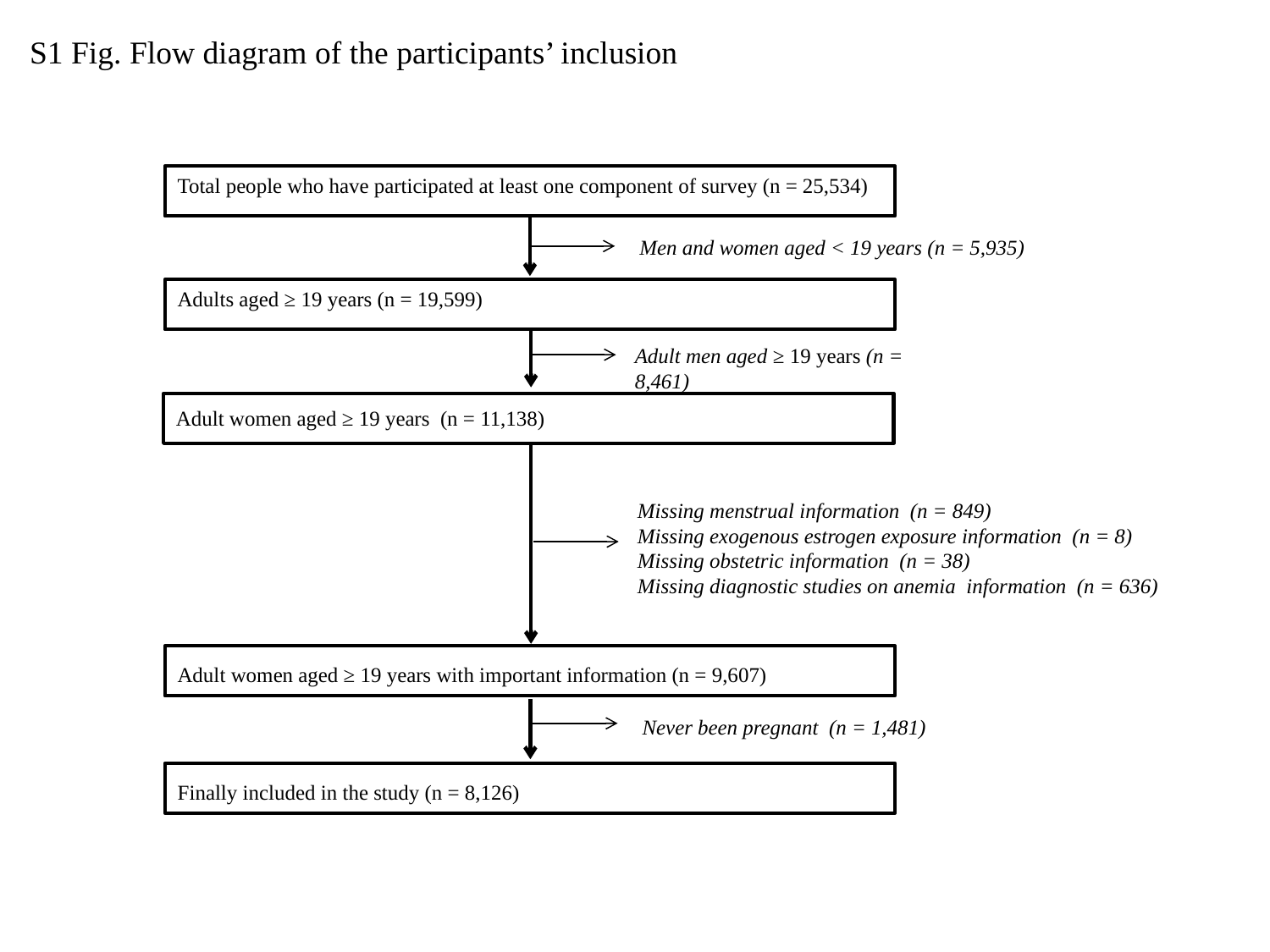

S1 Fig. Flow diagram of the participants’ inclusion
Total people who have participated at least one component of survey (n = 25,534)
Men and women aged < 19 years (n = 5,935)
Adults aged ≥ 19 years (n = 19,599)
Adult men aged ≥ 19 years (n = 8,461)
Adult women aged ≥ 19 years (n = 11,138)
Missing menstrual information (n = 849)
Missing exogenous estrogen exposure information (n = 8)
Missing obstetric information (n = 38)
Missing diagnostic studies on anemia information (n = 636)
Adult women aged ≥ 19 years with important information (n = 9,607)
Never been pregnant (n = 1,481)
Finally included in the study (n = 8,126)
